# Retention of Full Dentures*Read before the Chicago Dental Society Dec. 18, 1917, and abstracted from the *Dental Review* for March.

**Published:** 1918-04

**Authors:** Rupert E. Hall


### RETENTION OF FULL DENTURES*

BY RUPERT E. HALL, D.D.S.

The success of an artificial denture can be no better than its supporting foundation—the jaw. Hence, the accuracy with which we study and utilize the foundation may determine the success or failure of the restoration. Equal study and care, however, must be given the form and arrangement of the teeth, as the retention of the substitute may be impaired and the denture condemned

*Read before the Chicago Dental Society Dec. 18, 1917, and abstracted from the *Dental Review* for March.

as ill fitting and unsatisfactory; when the real trouble lies in faulty form and arrangement of the teeth. Thus do we find that the successful retention of artificial dentures is not wholly dependent upon good impressions, but upon the form and arrangement of the teeth as well.

The fundamental factors underlying and governing denture retention then, are form and accuracy of mechanical design of the restoration and the resultant physical forces retaining the substitute. Mechanical form and accuracy of design of the denture are first in importance and in requirement, because the physical is the resultant of the mechanical relations created between the denture, tissues and antagonizing teeth.

The theory of retention and the method of denture adaption of construction for its accomplishment, about which this paper deals in part, insures the maximum amount of retaining forces of adhesion, cohesion and atmospheric pressure.

The requirements of the construction are, that the base of the denture should cover the greatest surface possible and have added to it a periphery with border surface continuous with that of the base of the denture and that the surface of such border be extended upon and adapted to the flexible peripheral tissues so that there is created a seal and valve like action between the flexible tissues of the periphery, to preclude, therewith, the ingress of air under the base of the denture and resist or prevent complete dislodgement of the restoration through the indirectly applied resisting forces of the atmosphere should displacement occur.

Extent of jaw surface and degree of apposition between the base of the denture and the adapted surface of the jaw tissues determine the relative extent of the respective forces exerted by adhesion and cohesion retaining the artificial denture.

Peripheral construction and adaptation for a seal and valve like action with the flexible peripheral tissues, sealing space occurring between the base and jaw created by displacement of the denture, preventing the ingress of air, forming a relatively increasing evacuated space indirectly applying the force of the atmosphere thereby, aids in preventing or opposes partial or complete dislodgement of the artificial denture should displacement occur.

The ideal condition for the maximum utilization of the forces of adhesion, cohesion and atmosphere pressure for the retention of an artificial denture against displacement and partial or complete dislodgement, is to have the base cover the entire surface of the jaw; to add to the base a periphery, extending upon and adapted to the flexible peripheral tissues, sealing the peripheral border against the ingress of air.

Credit for the idea of constructing and establishing such relations between the base periphery, jaw and flexible peripheral tissues, in so far as our information is confirmed, should be given Dr. W. V-B. Ames, of Chicago, for it was he, in so far as we know, who, years ago, first conceived their importance and in 1885 (*Independent Practitioner*, July) demonstrated their principles. Others notable in early appreciation and use of their principles, were the Greene Brothers of Missouri, and especial admiration and appreciation should be held for their untiring, constant and persistent labors for their adoption. Credit belongs to these men, so we understand, for the correctable compound method. Also the excellent work of Mr. Samuel G. Supplee, of New York, on the technic in the use of modeling compound should not be overlooked.

The method of denture adaptation to be illustrated and described permits, it is believed, of an infinitely greater surface tissue adaptation and wider range of movements of the denture without causing displacement and unsealing or breaking of the peripheral valve seal, should displacement occur, than is secured in less accurate methods of adaptation or in the old method of denture construction where peripheral adaptation and valve seal are entirely absent and the edges of the denture permit the ingress of air, and complete dislodgement of the restoration should displacement occur.

To fully utilize mechanical and physical forces in the retention of artificial dentures, one should know exactly what jaw conditions favor and what conditions do not favor retention. To this end adoption of a classification of jaws and standardization of their requirements and technical procedure for the attainment of such requirements is, in the opinion of the essayist, not only desirable, but essential to a better understanding of denture construction and retention.

That edentulous jaws may be classified and divided into two general groups, is true, and that through such classification we shall be directed to a better understanding of the physics, mechanics and general conditions governing full denture retention is equally true. Also, that through such understanding definite lines of procedure may be formulated and each class rationally treated thereby.

Class I may be designated as that type of edentulous jaws that may be classed as normal—jaws with well defined ridges and normal quantity and tone of the overlying and adjacent tissues.

Class II may be designated as that type of edentulous jaws that are abnormal—jaws with poorly defined or excessively absorbed ridges, marked distortion or loss of facial contour and abnormal or subnormal quantity and tone of the overlying and adjacent tissues.

Class I jaws offer the greatest amount of surface for adaptation, consequently, greater physical forces for denture retention. The well defined ridges in this class also offer mechanical retention assisting in securing the denture against horizontal mobility, and often secure the restoration against vertical displacement. These, together with the ideal cushion support offered by the normal overlying and adjacent tissues, make Class I jaws easy of artificial denture retention.

Jaws of this class may be and are usually fitted with the most indifferent construction and adaptation, most any kind of an impression and denture construction serving to produce passable retentive results. Retention in this class is not so dependent upon peripheral construction and valve seal, nor is particular arrangement and efficiency of the teeth especially essential to passably satisfactory retention.

Class II jaws are *entirely dependent for retention upon the physical forces alone;* no mechanical retention of consequence is afforded in this class. Jaws of this class require for their successful management the closest adherence to every detail of the mechanical relations of the dentures to the tissues and to one another.

The greatest available jaw surface possible for base adaption should be utilized in this class. Peripheral construction and valve seal should be carefully and positively secured. And in case of the upper jaw, peripheral valve seal with the flexible tissues of the soft palate under considerable pressure, is strongly indicated. Insuring as nearly as possible in this the most difficult region for maintenance of adaptation of the periphery of the denture with the flexible tissues, a perfect valve seal against the ingress of air upon displacement and partial dislodgement of the denture.

The basic essential in the taking of a perfect impression for full and complete utilization of the jaw and flexible peripheral tissues desired to be utilized for the adaptation and retention of an artificial denture is a tray suited to the case. It is my belief that the only accurate and satisfactory way to procure a suitable tray, is to construct a special tray for each individual case.

### THE IMPRESSION MATERIAL

Plaster of Paris, mixed to the correct consistency, is far more yieldable and adaptable than any other material with which we are familiar, and by the aid of a correctly formed individual tray, may be handled with such control that any desired form of impression may be secured. The particular feature of "post-damming" or adaptation under pressure upon the flexible tissues of the soft palate, however, seems best accomplished with modeling compound.

### INDIVIDUAL TRAYS

For the construction of individual trays. use The S. S. White Impression-Tray Compound, which was suggested by me for the particular purpose of making individual trays quickly, efficiently and economically. It is jet black in color to make it readily distinguishable; has a high melting point to assure, when set or hardened, ample rigidity against distortion in removal from the metal tray and subsequent shaping and handling. Select a tray of the proper shape for the case, but somewhat larger than you would ordinarily use. Fill the tray with the compound, softened in hot water. Pass the exposed surface of the compound over a Bunsen or alcohol flame to remove inequalities in the surface and give it a glaze. Plunge into hot water to wet the surface and prevent sticking to the tissues, and as soon as it cools to a bearable degree insert in the mouth and secure a compound impression in the regular way. In a short time it can be removed from the mouth and placed in cold water to harden.

Remove the impression from the metal tray, and with a sharp knife, trim away the excess compound approximating the peripheral outline and contour of the proposed tray.

### TRIMMING THE UPPER TRAY

Beginning at the labial margin, the tray is trimmed thin at the labial frenum, and allowing liberal relief at the frenum. Passing on to the region formerly occupied by the cuspid teeth on either side, the tray is given a gradual fullness or prominence, restoring the cuspid eminences. The tray should be as high and full in this region as may be required to lift or displace the tissues for retention of the proposed denture and restoration of disturbed facial contour—the idea being to accentuate and build up the jaw ridge by increasing its area, and make in the finished impression the desired facial restoration.

Passing posteriorly from the cuspid eminences or about midway between the cuspid eminences and the tuberosities, we find the malar process of the maxilla, which registers a downward curve in the compound impression. The process is so situated that it ascends or descends at an angle of about 45 degrees to the general plane of the alveolar ridges or base of the proposed denture.

The margin of the tray in this region must be given particular consideration and preparation. The process is thinly covered with tissue and disposed at an unfavorable angle to permit of much vertical movement or bearing of the denture and undue pressure in this area should be certain.

Moving posteriorly of the malar process, a well defined cavity, as a rule, is found, and offers extremely valuable area for peripheral adaptation and denture retention. This space may be called the buccal cavity, and be defined as the cavity formed or bound by the malar process, the cheek, the angle of the mouth and the tuberosity. It is indeed amazing how little this valuable space is utilized, and on the other hand quite astonishing how extensively it may be utilized. In forming the tray for this space, allow it to go well up into the cavity, filling it buccally as well as vertically, preferring, however, to accentuate or favor vertical height rather than buccal fullness.

Next, outline and trim the posterior or palatal border of the tray. The outline of the tray should approximate that of the junction of the hard with the soft palate. Its length, however, should extend well upon the soft palate, the exact length of which will be determined in a later operation. Cut out the tray to relieve locks at undercuts, and areas of soft flabby ridge tissues allowing them to hang freely in the tray.

Construction of dentures for upper jaws not requiring facial restoration or permitting presence of base and periphery upon the tissues in the labial region of the jaw and flexible peripheral tissues, do not permit peripheral adaptation in the buccal areas under pressure upon the flexible peripheral tissues. Construction and adaptation of periphery upon the flexible peripheral tissues in the buccal areas are desirable, but care should be exercised in preventing pressure, the positive force thus created by displacing the tissues would react against retention of the denture and would not, in the absence of periphery and peripheral valve seal in the labial region, be met with and overcome by atmospheric pressure by the forming of an emergency vacuum upon displacement of the denture from its position of basal seat as in the case of that afforded where peripheral construction and valve seal are complete and perfect. This class of cases may be properly subclassed and would come under the subclass of Class I jaws.

### TRIMMING THE LOWER TRAY

The general preparation of the lower tray is the same as that of the upper. The lower jaw, however, has its individual characteristics. One is that absorption takes place in such manner that the curve or circumference of the ridge remains practically unchanged or fixed. Whereas, in the case of the ridge of the maxilla, absorption reduces its circumference quite extensively. Consequently facial contour is less disturbed in the loss of the lower teeth than in the upper. Therefore, less fullness is required in the lower denture for the restoration of disturbed facial contour than in the upper.

The lower jaw, like the upper, also has much overlooked and neglected valuable surface and flexible peripheral tissue surface for base and peripheral adaptation for denture retention. Aside from our general failure to utilize the available area, the lower, like the upper jaw, has, as a general rule, two spaces that are much overlooked. These may be called the lingual spaces. They lie on either side of the tongue and are bound by the mylohyoid ridge, the floor of the mouth and the tongue. These spaces are, when present, and utilized, valuable aids to denture retention in those excessively absorbed cases, or so-called flat jaws. Fit the tray well into these spaces, aiming to utilize them in the completed denture. The supporting foundation or ridge of the lower jaw is more or less circular back to the region of the first molars. The diverging flanges formed by carrying the base of the denture into these lingual cavities will act as tangents to the circle and prevent or assist in preventing, horizontal movement of the circular base.

The individual tray being approximately outlined, is now ready for final shaping and conformation to the tissues.

### SHAPING AND CONFORMING THE INDIVIDUAL TRAY

Shape and conform the tray to the tissues without undue pressure or fullness, but under just that degree of pressure and fullness as may be indicated and desired. There are, in my judgment, two acceptable ways of best conforming the periphery of the tray to the flexible tissues. One method is that of the late Dr. Greene, previously referred to, and is no doubt familiar to most of you. The method consists of tracing modeling compound upon the edge of the tray and while hot and plastic inserting the tray in the mouth and having the patient make movements of the muscles which in turn cause the compound to flow and conform to the tissues.

The method in its application to the upper jaw consists of successive layers of very thin plaster. As a rule only two mixes are necessary. The first mix registers the position and approximate extent of the imperfections of the improvised compound tray. Where the tray is too long or impinges, the plaster is displaced, and where too short, plaster is added. The tray is freed of excess and points of impingement are cut away to free the tissues impinged. The tray made up partly of tray compound and partly of plaster may now be considered perfect and is ready for the second mix of plaster with which we plan to secure an accurate impression of the jaw under such displacement and pressure upon the tissues as previously decided advisable and predetermined in the preparation of the individual tray.

Since adaptation under pressure upon the flexible tissues of the soft palate is a prerequisite to retention in its maximum degree; and since there is no means of confining or restricting the escape of the flowing thin plaster from the tray about the tissues in this region, as in the case of the labial and buccal borders where the tray is overlapped and bound in by the tissues of the cheeks and lip, it is obvious that adaptation of the palatal border of the denture under pressure upon the soft tissues must, if accurately made, be secured by means of some plastic material the flowing stress of which offers such resistance as may be required to give the desired pressure upon the tissue.

Modeling compound seems to be the ideal material for use in this connection.

The posterior border or length of the compound impression tray is cut off conforming in outline to that of the junction of the hard with the soft palate, trimming to such length as it is desired the finished denture should be.

Modeling compound, preferably Kerr's in stick form, is softened with dry heat and traced upon the top of the palatal surface of the black compound tray. The impression is next dipped into warm water to prevent the compound sticking to the tissues when it is inserted into the mouth and adjusted to place. Adjustment of the impression to its seat is made as the varying temperature and flowing resistance of the compound against the tissues being impressed may indicate to effect the required amount of pressure upon the flexible peripheral tissues of the soft palate as adjustment of the impression to its seat progresses.

Securing adaptation under pressure upon the tissues of the flexible soft palate by this or some equally scientific means, insuring tension of the tissues under equalized pressure is strongly advocated.

Pressure engagement of the periphery of the denture with the tissues by molding or swaging the base upon casts that have been altered by cutting and scraping to increase the extent of engagement of the periphery with the flexible peripheral tissues, is guesswork and unscientific. The frequent injury of the tissues and the suffering imposed by such practice evidence the empiricism of the method and warrant discouragement of its practice. Casts made from accurate impressions secured in accordance with the demands of the case require no cutting or scraping.

Careful study and outline of the hard palate should be made and generous relief of any pressure of the base of the proposed denture upon this area should be certain. Otherwise, pressure of the base upon the tissues of the hard unyielding area may establish a fulcrum, cause rocking of the base and impair the stability of the structure.

The greatest care should be given the selection and arrangement of the teeth in this class. Teeth with long, sharp, efficient cusps should be employed, that the forces necessary to the functions of efficient incision and mastication may be minimized. Careful study should be made of the arrangement of the teeth, with especial regard to levers that are formed under the stress of the forces of incision and mastication and tend to dislodge and impair the stability of the dentures. And lastly, but in no wise less important, is the anatomic relation of the teeth. These should be carefully worked out, their aid in increasing the efficiency and balancing the dentures in the process of incision and mastication determines in a very great measure the success of denture restoration.

### FORM AND ARRANGEMENT OF THE TEETH AS AN AID TO DENTURE RETENTION

### *Anatomical Occlusion and Articulation*

My understanding of the meaning of the term anatomical occlusion and articulation is, that the teeth, during movements of the mandible, when in the varying positions in the process of mastication, should be so arranged that the cusps of the teeth of the opposing jaws shall establish such relations of contact between them while gliding to the position of fixed occlusion, varying spaces should be formed to cut, crush and grind the food as the closure of the teeth progresses, providing, thereby, points of support against displacement of the dentures, by contact of the antagonizing cusps, with spaces for food.

If this be true, I want to go down in the record of the proceedings of this meeting as saying that, in my opinion, we have had, up to this time, no anatomical occlusion and articulation.

The use of the face bow; the measuring and recording of the condyle paths; the movements of the so-called anatomical articulators; the setting of teeth to theoretically determined planes and curves in wax to the arrangement known as three-point contact, have all been productive of nothing in assimilating anatomical occlusion and articulation. Therefore, no aid to denture retention through this supposed source has been an actual reality, and those advocates and followers of such theories and practice have, in my opinion, been running wild on a delusive theory.

These theories, in part, teach that the general plane of the teeth should be arranged, irrespective of the alveolar ridges, parallel with a line drawn from the ala of the nose to the tragus of the ear. This and its associate rules have long since been found wanting and have not been, nor to my mind will they ever be generally accepted. Although, from the voluminous literature upon the subject, and especially the work and claims of one of our manufacturers, one would be led to believe that the last word on the subject had been said and perfection of its practice attained. Few there are, comparatively, however, that have adopted and are practicing its teachings, and these few will, in time, like the rest of us, discover its shortcomings and abandon its practice. So we shall share the keenest pleasure in the disappearance from our text books and literature of these much overworked theories, that we may not be further misguided into error.

Do not be discouraged, however, for there is such a thing as anatomical occlusion and articulation, and when such form and relations of the teeth are secured, not only greater efficiency, but extremely valuable aid to denture retention results. It is my opinion that the form and arrangement of the teeth in Class II jaws, those requiring physical adaptation, are as important and share equally in importance with that of the construction and adaptation of the base and periphery of the denture.

What, then, is anatomical occlusion and articulation? How, and by what means, may we arrange teeth anatomically, so as to secure greater efficiency and their aid in the retention of artificial dentures?

Anatomical occlusion and articulation means that the artificial teeth should be so formed and arranged that they will occlude and articulate without undue interferences, balance and support the dentures against displacement under the stress of the forces of incision and mastication, guide the coordinating muscles in directing the movements of the mandible and at the same time, in the positions or relations of articulation, provide spaces for food.

Teeth may be anatomically arranged by having an articulator capable of imitating all of the movements of incision and mastication of the mandible and then have these movements under such control that we may utilize them for the perfect positioning and grinding of the teeth in anatomical relations as guided by the esthetics, the alveolar ridges and the movements of the articulator.

Present teaching and practices are now wrong. First, because of faulty methods and articulators. Second, because the cusps of the teeth now upon the market are not long enough to prevent meshing or interlocking so that in movements of the mandible they will reach and maintain contacts with their antagonists, provide adequate spaces for food and balance the dentures under the stress of the forces of mastication.

Teeth with deep angular cusps (guiding angle 45 degrees), correctly arranged, balance the vertical and lateral or horizontal movements of the mandible, as typical to nature, when in the stage of greatest efficiency; definitely guide the coordinating muscles in directing the movements of the mandible, also typical to nature; provide spaces for food; balance and support the dentures in the process of incision and mastication against movements from their basal seat; and in case of displacement of the dentures, steady and guide their return to their normal position comparable with the guiding and supporting influence of the rails of the railway track upon the railway coach, the rails the lower teeth (high cusps) and the wheels their flanges (long cusps) the upper teeth. The train without the flanges upon its wheels to secure and guide its movements upon the rails (movements of the mandible in the case of the masticatory apparatus) would leave the track, and not travel its intended course and be a failure. Likewise may we justly and logically regard the principles involving the construction and uses of artificial dentures if we are to expect them to work to perform the function of incision and mastication efficiently, and not be a failure. Especially restorations required in Class II jaws. We must, therefore, incorporate or provide in their construction suitably formed and correctly arranged teeth or they will not be guided and supported in the functionating movements of the mandible and will be a failure to such degree as they may be deficient in the requisite fundamentals to the highest and most efficient type of denture restoration.

In our latest edition of a prosthetic text book, we find and quote the following:

"Let us consider the relation of the mandible to the maxilla, first, with the natural teeth present and in occlusion, and second, after the jaws become edentulous. In the first instance the masticatory muscles bring the mandible upward until when the teeth are in occlusion it is in a state of rest. In this position the facial profile is normal, while the lips rest easily against each other without apparent muscular tension, or conscious effort on the part of the individual."

In the above quotation we note the author states that when the teeth are in occlusion, the mandible is in a state of rest. If there is anyone in this audience whose mandible, when the teeth are occluded, is in a state of rest, please make the fact known in the discussion following this paper. It will be the first one I have ever known.

The teeth are occluded through muscular effort only, therefore the mandible could not, in the position of occlusion of the teeth, be in a state of rest.

We note further that when the mandible is in this position (of occlusion of the teeth), the facial profile is normal, while the lips rest easily against each other without apparent muscular tension, or conscious effort on the part of the individual.

Since the normal facial profile is that of the features when the muscles are relaxed and in a state of rest; and since it is true that, normally, the mandible is not in a state of rest when the teeth are in occlusion, we must conclude that the rule of determining the length of the bite and subsequent positions of the proposed teeth according to the above indications, are not esthetically or anatomically correct.

The present rule is to set the teeth to approximate a length in line with the lips and not overlap the upper or under-bite the lower anterior teeth to any considerable degree, owing to leverages that would be formed, displace the dentures and break off the anterior teeth.

This would be a very fine rule if turned the other way around. The rule is absolutely wrong and its application is the greatest cause for displacement of dentures and breakage of anterior teeth. Why? Because, to set the anterior teeth in line with the lips and not permit them, when occluded, to pass or overlap as in nature, means simply this, that the molars and bicuspids have to be raised and lowered to occlusion to a bite corresponding in distance between the ridges with the incisive bite, instead of the occluded bite. In other words, following this rule produces dentures the presence and operation of which in the mouth force the bite open to the extent of the depth of normal overbite and interfere with the movements of the mandible, and the patient experiences a feeling that the mouth is held open and the teeth are too long. Therefore, teeth so arranged do not efficiently restore the function of incision and mastication, and the excessively long molars and bicuspids interfere in the movements of incision and mastication, dislodge and impair retention of the dentures.

In the absence of overlapping anterior teeth, as normal to nature and necessary to the function of incision, the patient manages to find a point of contact between two of the opposing end to end related incisors and in their effort to incise, it is discovered that the teeth do not slide by and shear the object attempted to be incised, but merely pinches or punctures it, and in this predicament the patient thoughtlessly, though naturally, pulls the object in two, with the frequent result of a broken tooth.

The incisors, if correctly arranged to overlap, and ground to establish perfect opposing articulating edges and planes, will, with the minimum amount of pressure, efficiently perform the function of incision and equally distribute the pressure upon all the anterior teeth. And in so arranging them the molars and bicuspids will be relatively shortened and normal anatomical relations restored.

Teeth should be so arranged that they conform to the esthetic, anatomic and physiologic needs of the patient.

The misleading and absurd rule that confines us to a definite line of procedure in obtaining the relation the teeth and jaws should bear to each other, irrespective of the modifying influences of the individual patient, should in my opinion, be disregarded altogether.

Each case of denture construction is a law unto itself and no rule of averages, be it ever so inclusive, can possibly supplant or adequately supply the specific demands arising in the individual case. The rule that the length of the teeth should be established in accordance with the lips when in position of repose, is no less impractical than is the rule of establishing the occlusal plane.

The misleading feature of this rule, by which we are supposed to determine the length of the teeth, is due entirely to the fact that the rule does not conform to the conditions imposed in nature. When the muscles controlling mandibular movements are in a state of rest the lips normally are extended to their full length. The muscles controlling mandibular movements are not constantly contracted—nature could not tolerate a constant contraction of these muscles—and in their unfunctionating state the mandible hangs down to the extent of relaxation of the suspending muscles and tendons and the teeth are not normally in occlusion. The reverse of this normal repose in lip position would be found when the teeth are occluded and the lips are compressed and much shortened. It is my idea that the latter position of the lips more nearly indicates the length of the bite when the teeth are in occlusion than does the position of the lips when the muscles are relaxed and at rest.

I have dealt at length on this particular point because it will help to emphasize the things I am contending are wrong in respect to some of the rules and theories heretofore set down as definite facts, which, in my judgment, are very great hindrances to our success and advancement in the art of denture construction, both as regards retention and efficiency. Grinding the teeth is a necessary step in all cases, and is accomplished by applying carborundum to the teeth in the Automatic Anatomic Articulator. And if the teeth occlude and articulate in the mouth the same as in the articulator, either after one or more grindings, it proves the correctness of the movements of the articulator.

As to the rectangular spaces for food displayed upon the screen, formed by the teeth in masticatory movements, to which you took exception, I insist they are true to nature. This has been amply demonstrated and is substantiated by others experienced in the application of the principles laid down by me and worked out in my articulator. I have never inserted a set of dentures that the patient did not instantly, in masticatory movements of the mandible, establish such relations between the teeth, the rectangular food spaces always being present.

With reference to the registration and recording of the condyle paths which Dr. Prothero states he considers the most important step in the arrangement of the teeth, I will relate an incident that disproved the importance of this theory. I constructed for the experts of the S. S. White Dental Manufacturing Company to disprove this condyle path theory and to prove the logic of my articulator, three sets of dentures for the same individual, with varying arbitrary condyle paths. One set with paths of 24 degrees, one with 32 degrees and the other paths of 45 degrees—all with teeth of corresponding guiding cusp angles. Without regard to masticating efficiency, all worked perfectly, either set working as well as the other. The real truth of the matter is, that the extent of the movements of the condyles in masticatory movements proper are so infinitesimal that their true course and extent of movement is inconsequential. Therefore, let us desist from further study and attempts at recording their movements. There is nothing to the theory, as interpreted, and still less to the practice. The teeth guide the masticatory movements of the mandible and not the condyles.

Faulty form and arrangement of the teeth will cause dislodgement of the dentures. They may interfere or through inefficient cutting and grinding edges and planes require greater pressure or force for the perfect incision and mastication of food than the mechanical and physical retention of the bases will counteract. The entire paper and illustrations were aimed and it is believed do relate directly to the subject. It is feared that adaptation of the base to the jaw has been too much looked upon as the lone factor governing the retention of artificial dentures whereas the facts are, this is but one factor in the retention of artificial dentures.

